# Cancer Immunotherapy and Application of Nanoparticles in Cancers Immunotherapy as the Delivery of Immunotherapeutic Agents and as the Immunomodulators

**DOI:** 10.3390/cancers12123773

**Published:** 2020-12-15

**Authors:** Tilahun Ayane Debele, Cheng-Fa Yeh, Wen-Pin Su

**Affiliations:** 1 Institute of Clinical Medicine, College of Medicine, National Cheng Kung University, No.138, Sheng Li Road, Tainan 704, Taiwan; z10803012@ncku.edu.tw (T.A.D.); u802091@gmail.com (C.-F.Y.); 2 Department of Internal Medicine, Chi Mei Medical Center, Tainan 710, Taiwan; 3 Departments of Oncology and Internal Medicine, National Cheng Kung University Hospital, College of Medicine, National Cheng Kung University, Tainan 704, Taiwan

**Keywords:** cancer, cancer immunotherapy, nanoparticles, immunotherapeutic agent, immunomodulators

## Abstract

**Simple Summary:**

Cancer becomes one of the major public health problems globally and the burden is expected to be increasing. Currently, both the medical and research communities have attempted an approach to nonconventional cancer therapies that can limit damage or loss of healthy tissues and be able to fully eradicate the cancer cells. In the last few decades, cancer immunotherapy becomes an important tactic for cancer treatment. Immunotherapy of cancer must activate the host’s anti-tumor response by enhancing the innate immune system and the effector cell number, while, minimizing the host’s suppressor mechanisms. However, many immunotherapies are still limited by poor therapeutic targeting and unwanted side effects. Hence, a deeper understanding of tumor immunology and antitumor immune responses is essential for further improvement of cancer immunotherapy. In addition, effective delivery systems are required to deliver immunotherapeutic agents to the site of interest (such as: to Tumor microenvironments, to Antigen-Presenting Cells, and to the other immune systems) to enhance their efficacy by minimizing off-targeted and unwanted cytotoxicity.

**Abstract:**

In the last few decades, cancer immunotherapy becomes an important tactic for cancer treatment. However, some immunotherapy shows certain limitations including poor therapeutic targeting and unwanted side effects that hinder its use in clinics. Recently, several researchers are exploring an alternative methodology to overcome the above limitations. One of the emerging tracks in this field area is nano-immunotherapy which has gone through rapid progress and revealed considerable potentials to solve limitations related to immunotherapy. Targeted and stimuli-sensitive biocompatible nanoparticles (NPs) can be synthesized to deliver immunotherapeutic agents in their native conformations to the site of interest to enhance their antitumor activity and to enhance the survival rate of cancer patients. In this review, we have discussed cancer immunotherapy and the application of NPs in cancer immunotherapy, as a carrier of immunotherapeutic agents and as a direct immunomodulator.

## 1. Introduction

Cancer becomes one of a killer disease and its burden is anticipated to increase worldwide due to population growth, and lifestyles changes (such as smoking, poor diet, physical inactivity) [1,2]. According to global cancer observatory data (GLOBOCAN), 9.6 million deaths from cancer were estimated in 2018 [3]. The widely known conventional treatment methods for cancer include surgery, chemotherapy, and radiotherapy [4]. Due to the increasing knowledge of molecular and cancer biology, a notable change was observed in cancer treatment for the last few decades. However, conventional cancer treatment has certain limitations, which urges further research investigation. Recently, different research has been underway to improve the survival rate of cancer patients which includes immunotherapy, stem cell transplantation, and targeted cancer therapies [5,6,7,8,9,10].

Herein, we briefly discuss the application of nanoparticles (NPs) in the cancer immunotherapy as the carrier of immunotherapeutic agents and as the adjuvants to stimulate immune systems to eradicate cancer.

## 2. Nanoparticles and Nanoparticles-Based Drug Delivery Systems

The majority of drugs delivered through a different route of injection, encounter the physiological, biochemical, and chemical barriers [11]. Hence, it is important to know the physicochemical and biochemical nature of the pharmaceutical agents such as solubility, permeability, and metabolic stability which are crucial factors in the design of NPs for drug delivery systems [12]. In comparison to conventional drug formulation, NPs-based drug delivery systems are under extensive development for several applications including cancer treatment due to their unique physical, chemical, and structural properties. In the last few decades, the term nanomedicine is popularized to describe the application of nanotechnology, by exploiting the unique properties of nano-scale materials, in medicine for the diagnosis and treatment of disease.

Tumor blood vessels possess special characteristics in comparison to the normal blood vessels such as uncontrolled angiogenesis, aberrant vascular architecture, hypervascular permeability, and impaired lymphatic clearance from the interstitial space of tumor tissues (i.e., enhanced permeability and retention (EPR) effect) [13,14]. EPR effect is a crucial point in the drug delivery systems [15,16]. Several kinds of the literature showed that NPs with the diameter 10–100 nm in the bloodstream are too large to escape the vasculature and enter normal tissues or to be cleared by the kidneys, while NPs can easily escape and accumulate in the tumor tissues due to dysfunctional vasculature and defective lymphatics clearance [17].

The efficacy of nanoformulated pharmaceutical agents also determined based on NPs characteristics such as sizes, shapes, and surface charge [18,19]. As mentioned above, NPs with a diameter range of 10 to 100 nm are the best candidates for cancer therapy, as they can effectively deliver their cargo and achieve EPR effect, while NPs with smaller (<10 nm) and larger particle size (>200 nm) can be easily filtered by kidneys and phagocytosed by reticuloendothelial systems, respectively [20]. However, failures of NPs-based chemotherapy in clinical trials have raised some questions about the clinical relevance of the EPR effect and much more research investigation is required to understand the tumor microenvironment (TME). In addition, ligand-modified NPs are widely explored for the active tumor targeting that can enhance bioavailability and selective tumor accumulation which in turn enhance the therapeutic efficacy while reducing normal cytotoxicity.

Moreover, shape and surface charge are crucial in cellular uptake and bio-distribution of NPs. For example, unlike spherical NPs which vulnerable to protein adsorption, non-spherical NPs show less protein adsorption and prevent non-specific cellular phagocytosis which extends their stability and half-life in circulation [21]. Another important parameter is the surface charge of NPs which has a great effect on cellular uptake and in the induction of immune response. For example, cationic NPs show good transfection effects, and have a lysosomal escape tendency which helps to release cargo in the cytoplasm or other subcellular organelles [22]. However, due to their cationic nature, they adsorb more negatively charged serum proteins which hinders their bioavailability [23,24]. As the result, NPs are coated with hydrophilic materials such as polyethylene glycol (PEG), or polysaccharides such as dextran to minimize protein corona, which in turn enhance circulation half-life and its bioavailability [25,26,27].

NPs-based drug delivery shows a promising result in preclinical and clinical studies. Currently, approximately 50 nanopharmaceuticals agents are approved for cancer and other disease treatments by US FDA [28,29,30]. However, some nanomedicine products that have undergone extensive clinical trials were later withdrawn due to efficacy or safety concerns e.g., superparamagnetic iron oxide formulations Resovist and SINEREM [31,32].

### 2.1. The Application of Nanoparticles in Cancer Immunotherapy

The idea of cancer immunotherapy is boosting the antitumor activity of immune systems via tumor-specific immune activation or non-specific immune activation [4,33,34,35,36]. The cancer immunotherapy can be boosted via: (a) Increasing antigens presentation and induce specific cytotoxicity T-lymphocytes (CTLs) activity [37]. Naive CD8^+^ T cells activated and induced antitumor immune response when their receptors recognize antigens presented by Antigen Presenting Cells (APCs) (such as DC) in the context of MHC-I molecules [38,39]. Activated CTLs secrete several cytokines such as interferon-gamma (IFN-γ), tumor necrosis factor-alpha (TNF-α) and the crucial cytolytic mediators (perforin, granzyme, etc.), which improve antigen presentation and mediate anti-tumor effects [40]. (b) Guiding T-cells to the tumor using a bispecific Antibody (bAbs). bAbs offers a unique opportunity to redirect specific immune effector cells to kill cancer cells [41,42]. bAbs can bind simultaneously two different antigens or epitopes to guide T cells to tumor cells, to inhibit two different signaling pathways, and to deliver cargos to the targeted sites [43]. (c) The downregulation of Treg cell, or MDSCs. The TME is enriched with cellular and acellular components that negatively influence cancer immunotherapy [44,45]. MDSC and Treg cells are major components of the immune-suppressive TME and promote T-cell dysfunction that in turn favors tumor progression [46,47]. Hence, downregulation of Treg cell or MDSCs via administration of specific antibodies for each cell is crucial in cancer immunotherapy [48,49,50,51].

Immunotherapy offers numerous advantages in comparison to the conventional standard cancer treatment available nowadays [52,53]. Of those, when appropriately stimulated, tumor-specific immune cells can target a microscopic disease, disseminate metastasis, and long-term control might completely remove cancer due to the memory cells [54,55,56].

Although immunotherapy is efficient to treat different types of cancer, still there is a certain challenge in delivery of immunotherapeutic agents which is expected to be resolved using NPs [57]. A major goal of the utilization of NPs in cancer immunotherapy is to improve therapeutic index by enhancing deliver of immunotherapeutic agents directly to the site of interests only, enhancing accumulation and potency at a region of interest, while simultaneously minimize the dose-dependent systemic toxicity [58]. Unlike delivering chemotherapeutic agents to tumor cells, which necessitates a high dose of nanoformulated drugs to kill all the target cells to be effective, lower concentrations of immune-stimulating drugs can be used to initiate an immune cell or organs (such leukocytes or lymphoid organs) response [59]. For example, Schmid et al. developed antibody-targeted NPs that bind to CD8^+^ T cells in the blood, lymphoid tissues, and tumors of mice [60]. Synthesized NPs encapsulated with a SD-208, TGFβR1 inhibitor, or a TLR7/TLR8 agonist. Both in vitro and in vivo mice studies showed, successful targeting of PD-1^+^ T cells in the circulation and in the tumor. Compared to the free drugs, NPs-encapsulated SD-208 enhances survival of mouse bearing colorectal cancer. In addition, synthesized NPs enabled PD-1-targeted delivery of a TLR7/8 agonist to the TME and tumor-infiltrating CD8^+^ T cells were increased. Overall, this result shows that targeting tumor-infiltrating immune cells in the blood, rather than direct tumor cell targeting, is a better way to improve immunotherapeutic localization in tumors and to stimulate an antitumor response.

In summary, NPs based cancer treatment has a numerous advantage compared to conventional cancer therapy due to: (1) Nanoscale size with several surface characteristics to enhance drug accumulation at the site of interest via EPR effects, (2) Target tumor cells via active targeting which will minimize off-target normal cell toxicity, (3) Protect a therapeutic payload (such as protein, gene, small peptide) from biological degradation, (4) Enhance solubility of hydrophobic drugs and improves there bioavailability, (5) enhance in vivo stability and bioavailability, (6) prevents premature drug release, (7) used as theranostic, combined imaging and therapeutic applications and (8) stimuli, internal or external, programmed to release its cargo at the site of interest.

Furthermore, due to their effectiveness at eliciting cellular and humoral immune responses, NPs can be designed to activate the immune system that could form a gorgeous basis for cancer vaccine development [61,62]. As the result, several NPs are synthesized to deliver different types of immunotherapeutic agents to enhance their therapeutic efficacy, and some of them already shown satisfactory results in clinical trials [63].

#### 2.1.1. Nanoparticles as the Carrier of Immunotherapeutic Agents

**Figure 1:**
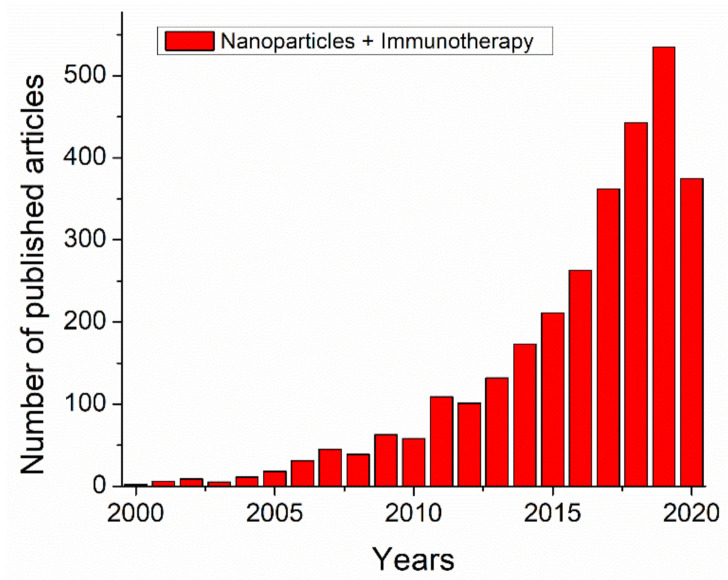
A number of published papers for the last two decades (i.e., 2000–2020) by searching on PubMed using key words “nanoparticles + immunotherapy”.

Over the last few decades, numerous studies and a large number of papers (Figure 1) have been published on nano-based therapies for cancer treatments. In the last two decades, the total number of papers related to ‘nanoparticle + immunotherapy’ on PubMed approximately doubled every two years which will be expected a rise similarly in the future.

NPs should be precisely designed to target region of interest preferentially from site of administration (common vaccine administration routes are mucosal or parenteral) in order to enhance the efficacy of immunotherapeutic agents [64,65]. NPs targeting lymphoid tissues, where the majority of immune cells are concentrated, would enhance the efficacy of immunotherapeutic agents due to direct access to immune cells [66,67,68,69].

Depending on their physicochemical characteristics, including particle size, hydrophobicity, shape, and surface charge, NPs can directly drain to the nearest lymph node, or stay in the injection site and attract migratory DC or macrophages [70]. Several kinds of the literature show that NPs with particle sizes > 100 nm tend to form depot and taken up by APCs and then draining to lymph nodes [71,72]. However, NPs with moderate particle size < 100 nm drained to lymph nodes via lymphatics and retained relatively for a long time, while NPs with small particle size (<10 nm) drain to blood capillaries [73,74]. Regarding the surface charge, negatively charged NPs drain to the lymph node was reported due to charge repulsion with negatively charged ECM, while, cationic NPs tend to form a depot, taken up by peripheral and migratory APCs or gradually draining to lymph node [75]. NPs with nearly neutral charge exhibited potential vector for tumor antigen because they targeted the draining lymph nodes after subcutaneous injection. However, they were weakly immune-stimulatory. In addition, the presence of PEG (PEGylation) on Surface of NPs significantly enhanced large particle size (~200 nm) drain to lymph node and uptake by DCs [76].

Moreover, targeted and stimuli sensitive biocompatible NP can be synthesized to deliver immunotherapeutic agents’ in their native conformations to increase antigen uptake, processing, and presentation. For example, NPs can be synthesized for facilitating the cytosolic delivery of antigens, increasing cross-presentation via the MHC-I pathway, and thus inducing cytotoxic T-cell responses. In addition, in the drug delivery system, it is possible to load or conjugate two or more than two drugs in the single nanocarriers as the co-delivery which will minimize dose related toxicity and enhance the activation of the immune response. For example, Song et al. developed the combined delivery of immunogenic chemotherapy and PD-L1 trap fusion protein using liposomal NPs [77]. They reported that PD-L1 trap is produced transiently and locally in the TME and oxaliplatin (OxP) boosts anti-PD-L1 therapy against murine colorectal cancer and exhibited reduced toxicity compared with non-nanoformulated ones (i.e., free PD-L1 antibodies and oxaliplatin).

In general, NP based drug delivery systems encompass a wide variety of nano-scale size materials including inorganic and Organic NPs in different forms [16]. 

#### 2.1.2. Antigens and Adjuvants Delivery to Antigen Presenting Cells (APCs)

APCs used as the link between innate and adaptive immune responses by interacting with T cells [78]. APCs are primarily used to recognize and present tumorigenic antigens on their surface via MHC complexes to T cells to initiate an effective adaptive response [79]. However, due to enzymes susceptible of antigens in the body, they are not easily transferred to APCs which decreases its immunogenicity. Hence by using NPs it is possible to overcome this limitation. NPs can encapsulate and deliver cancer antigens to APCs without tumor antigen degradation by the intracellular enzyme. In addition, nanoformulated antigens are more efficiently taken up and processed by APCs than soluble vaccines to amplify T-cell responses due to intradermally or subcutaneously injected NPs drain to lymph nodes, in which APCs are in closeness to T cells [80].

Shen et al. encapsulated ovalbumin (OVA) antigen in the PLGA and deliver successfully to primary mouse bone marrow-derived dendritic cells (BMDCs) [81]. Their result showed that the MHC class I presentation of PLGA-encapsulated OVA stimulated T cell IL-2 secretion at a 1000-fold lower concentration than soluble antigen and 10-fold lower than antigen-coated latex beads.

**Figure 2:**
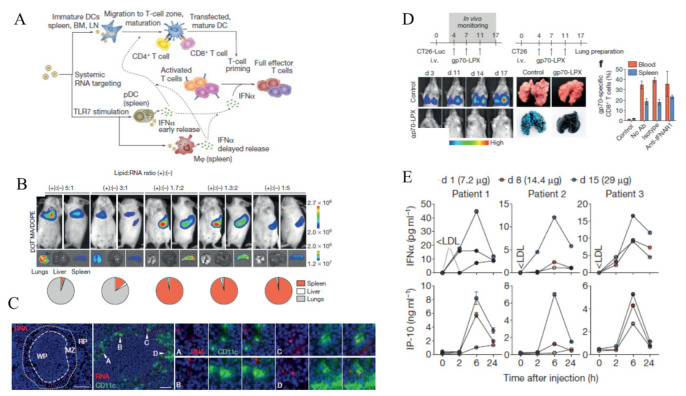
RNA-lipoplexes (RNA-LPX) delivery to DCs. (**A**) Mechanism action of RNA-LPX to induce anti-tumor immune responses, (**B**) Bioluminescence imaging of BALB/c mice, (**C**) Splenic localization of CD11c and Cy3 double-positive cells in BALB/c mice after 1 h of Cy3-labelled RNA-LPX i.v. injection, (**D**) in vivo studies in CT26 tumor bearing BALB/c mice immunized with gp70-LPX, and (**E**) Clinically administered RNA-LPX vaccines induce systemic INFα in dose-dependently manner. Reproduced with permission from [82]. Copyright 2016, Springer Nature.

Kranz et al. precisely designed RNA-lipoplexes (RNA-LPX) NPs with the particle size of ~200–320 nm, by optimally adjusting lipid: RNA ratio to precisely target DC using intravenous injection (Figure 2) [82]. The lipoplexes protect antigen-encoding RNA degradation by ribonucleases. In addition, RNA-LPX enhances cellular uptake and expression of the encoded antigens. Moreover, two transient waves of IFN-α were observed after the NP vaccine injection that led to better T-cell responses and produced vigorous and long-term antitumor effects.

Several kinds of literature showed that the targeted delivery of NPs formulated antigen into DC would enhance antigen presentation to T cells [83,84,85,86]. For example, Cruz et al. designed Pegylated PLGA NPs functionalized with TLR3/7 ligand to encapsulate OVA to target surface receptors of DC (i.e., CD40, DEC-205, and CD11c) to accomplish an effective cytotoxic T cell response [87]. In vitro cellular uptake study showed that TLR3/7 ligand targeted NP was more taken up by DC compared to non-targeted NP. Furthermore, high expression of IL-12, IFN-γ, and co-stimulatory molecules were observed in the ligand targeted NP in comparison to non-targeted NPs. Moreover, in vivo vaccination studies showed that ligand targeted NP consistently showed higher efficacy than non-targeted NP in stimulating CD8^+^ T cell responses.

Some research finding shows that immune response will be enhanced by co-delivering of adjuvants along with tumor antigens due to efficient antigen cross-presentation and vigorous T-cell response for tumor immunotherapy [88,89,90,91]. For example, Kuai et al. synthesized high-density lipoprotein-mimicking nanodiscs for co-delivery of CpG adjuvant and neoantigens [92]. They reported that, synthesized nanodiscs elicited up to 47-fold greater frequencies of neoantigen-specific CTLs than soluble vaccines and 31-fold greater antigen-specific T-cell response compared to Montanide. Moreover, nanodiscs in combination with anti-PD-1 and anti-CTLA-4 therapy revealed better eradication of established cancer cells.

**Figure 3:**
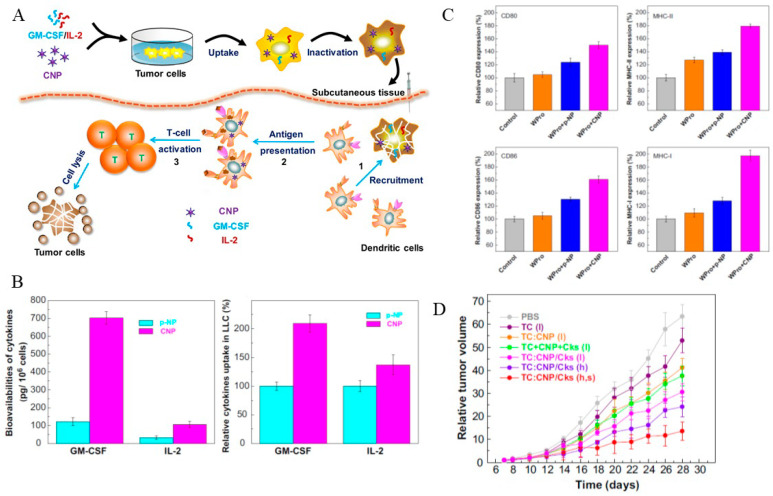
(**A**) Scheme of multi-adjuvant WCTV to initiate anti-tumor immunity, (**B**) bioavailability and cellular up take of GM-CSF and IL-2 in LLC cells after incubating with nanoparticles (NPs) for 24 h, (**C**) relative expressions of CD80, CD86, MHC II, and MHC-I molecules after treatment with whole tumor cell lysate protein (WPro), p-NP, and CNP for 24 h and (**D**) Relative tumor volume of LLC tumor bearing mice after immunization with multi-adjuvant WCTVs compared with other vaccine groups. Reproduced with permission from [93]. Copyright 2013, Elsevier Ltd.

Similarly, Liu et al. have been synthesized cell-penetrating peptide (CPP) decorated uniform-sized pristine NPs to deliver GM-CSF and IL-2 into tumor cells [93]. In vitro and in vivo (Figure 3) results revealed the programed promotions of multi-adjuvants on DC recruitment, antigen presentation, and T-cell activation. Furthermore, in vivo assessments revealed the satisfactory effects on tumor growth suppression, metastasis inhibition, and recurrence prevention.

#### 2.1.3. Antigens and Adjuvants Delivery to Tumor Microenvironment (TME)

The TME comprised proliferating tumor cells, the tumor stroma, infiltrating inflammatory cells, apoptotic cancer cells, cancer-associated fibroblasts, myeloid-derived suppressor cells, tumor-associated macrophages, and a variety of associated tissue cells which are participating in the suppression of antitumor immunity [94]. These immunosuppressive cells secrete numerous soluble mediators including, Transforming growth factor-beta (TGF-β), Indoleamine 2,3-dioxygenase (IDO), arginase, prostaglandin E2 and nitric oxide synthase 2 (NOS2) [95,96,97]. By reducing the supply of indispensable amino acids (such as arginine (R) and Tryptophan (W)), IDO and arginase directly suppress T cell proliferation and differentiation [98]. The activity of arginase and IDO translates not only into amino acid deprivation but also in the production of metabolites (such as l-kynurenine and spermidine) capable of numerous physiologic effects [99,100]. For example, l-kynurenine derived from W, favors the differentiation of Treg cells and induces IDO expression in DCs. Similarly, TGF-β also alters activation, maturation, and differentiation of DCs, CD4^+^, and CD8^+^ T cells. In addition, PD-L1/PD-L2 expressed on tumor cells can engage PD-1 receptor on the surface of activated T cell and sends inhibitory signals via activating phosphatases, resulting in dephosphorylation of key elements in the T cell, leading to down-regulating proliferation, survival, and cytokine production [101]. Furthermore, the CTLA-4 receptor on tumor cells binds to co-stimulatory molecules on DCs and decreases antigen presentation. Moreover, there are an abundant accumulation of acellular components such as fibrosis, collagen, secreted protein acidic and rich in Cysteine (SPARC), and hyaluronan which alters the physicochemical properties of TME (including physical barriers, physical pressure (i.e., increase interstitial fluid pressure, change in metabolism, etc.) [102].

As mentioned above, although several immune effector cells are recruited to the TME, their anti-tumor activity is suppressed principally in response to tumor-derived signals [103]. Compared with normal tissue, TME has some unique characteristics, such as vascular abnormalities, hypoxia, increases in proteolytic activity, and an acidic microenvironment, which leads to treatment resistance [104]. Therefore, new approaches are demanded to overcome TME related immunosuppressive situations. Hence, targeting immunosuppressive cells (such as Treg) or Tumor-associated macrophage (TAM) in the TME using NPs could be the best tactics to prevent immunosuppression.

Sacchetti et al. designed ligand guided PEG-modified single-walled carbon nanotubes (PEG-SWCNTs) to target Treg-specific receptors in the TME [105]. They found that ligand targeted PEG-SWCNTs were preferentially up taken by Treg cell residing in the TME via glucocorticoid-induced TNFR-related receptor (GITR).

Similarly, Zhu et al. synthesized mannose targeted PEG-sheddable NPs to target TAM [106]. They reported that mannose-modified PEG-sheddable NPs was effectively targeted TAMs via the mannose-mannose receptor. As a result, more PEG-sheddable NPs accumulation was observed in TAM in comparison to non-sheddable PEG. This is maybe due to PEGylation which minimizes NPs opsonization and enhances its bioavailability.

**Figure 4:**
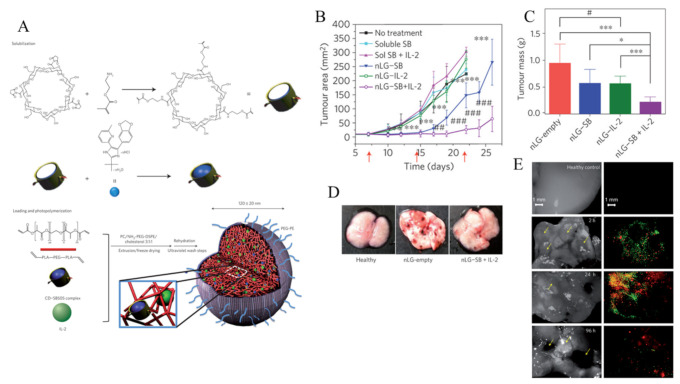
(**A**) The synthesis approach of the liposomal polymeric gel (nLG) particle system. (**B**) Plot of tumor area versus time. Red arrows indicate treatments (via intratumoral injection). (*p* < 0.05, *, *p* < 0.001, ***, By ANOVA with Turkey’s multiple comparison test. *p* < 0.05, #, by two-tailed t-test. (**C**) Tumor masses vs nLG-treated groups, *p* < 0.001, ***, *p* < 0.01, **, *p* < 0.05, *, By ANOVA using Turkey’s post-test. (**D**) Images of lung immediately before collection of lung-infiltrating lymphocytes from mice, (**E**) Uptake of lipid carrier (green) and rhodamine payload (red) around individual lung tumors at 2 h post injection. Reproduced with permission from [107]. Copyright 2012, Nature Publishing Group.

In addition, NPs can be used to deliver anti-immunosuppressive factors, such as anti-TGF-β or TGF-β receptor inhibitor to the TME to increases the activation of the immune system. Park et al. synthesized liposomal polymeric gels (nLGs) to deliver IL-2 and TGF-β inhibitors (Figure 4) [107]. The author reported that IL-2 and TGF-β inhibitors were successfully delivered to the TME. In vivo results showed that nLGs treatment suppresses a tumor growth, improved survival rates, and enhanced the activity of NK cells and intratumoral-activated CD8^+^ CTLs.

In summary, NPs can enhance anticancer immunity by regulating the TME either by inhibiting immunosuppression or by endorsing immune activation which could synergize with clinically established immunotherapeutic agents such as Immune Checkpoint Inhibitors (ICIs). Hence, targeting immune cells in the TME using nanoformulated therapeutic agents is the best tactic to activate antitumor immunity.

#### 2.1.4. Immune Checkpoint Inhibitors (ICIs) Delivery

Immune checkpoints are surface proteins on immune cells that act as negative regulators of immune activation by various antigens, including tumor antigens [108]. Immune checkpoint molecules include PD-1, PD-L1/2, CTLA-4, T-cell immunoglobulin and mucin domain-containing-3 (TIM-3), and lymphocyte-activation gene 3 (LAG-3) [109,110]. Immune checkpoint molecules are widely expressed on both tumor cells and immune cells, which might be negatively regulated by tumor-specific T cells via receptor–ligand interactions, causing T-cell anergy or exhaustion [111,112]. Tumor cells evade destruction from the immune system by triggering immune checkpoint receptors, such as CTLA-4, PD-1, or PD-L1, that are expressed on T-cells and whose engagement inhibits T-lymphocyte function [113].

**Figure 5:**
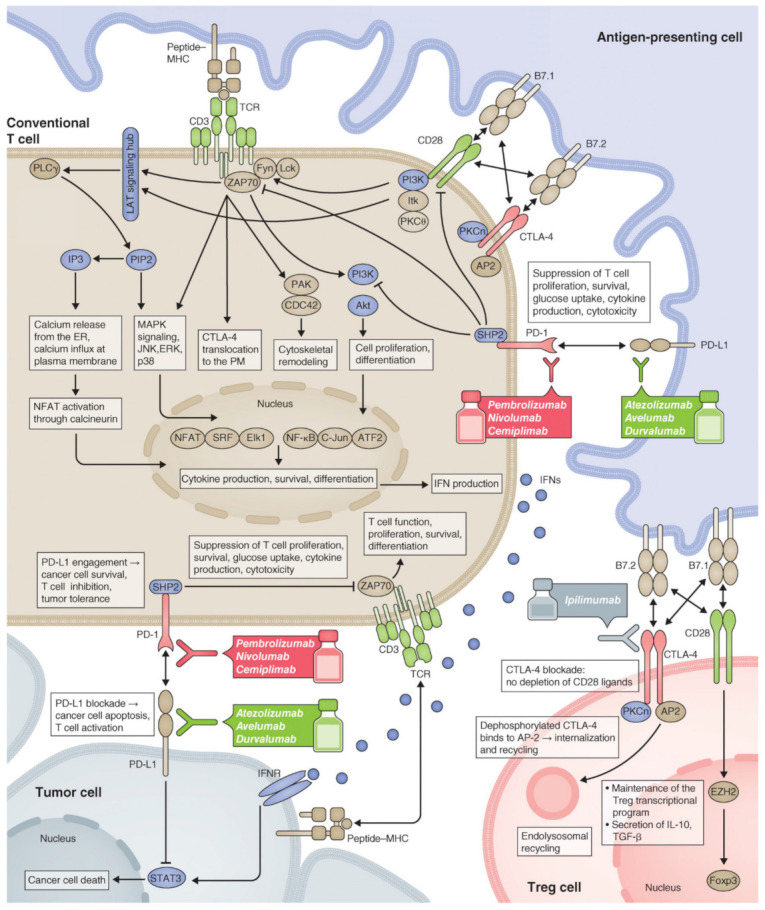
Mechanism action of Immune Checkpoint Inhibitors (ICIs). Reproduced with permission from the Journal of Cell Biology [124].

ICIs are monoclonal antibody that inhibits the receptors-ligands interaction and enhance immune-mediated cancer eradication. The development of ICIs lays a key foundation in cancer immunotherapy [114]. In 2018, James P. Allison and Tasuku Honjo were awarded a Nobel prize in physiology or medicine for the discovery of cytotoxic T-lymphocyte-associated antigen (CTLA-4), and programmed cell death protein 1/programmed cell death protein ligand 1 (PD-1/PD-L1), respectively [115]. According to literature report, anti-CTLA-4 antibody overcomes a block in essential costimulatory signals (i.e., CTLA-4 and CD28 competes for the same ligands CD80 and CD86; CTLA-4 has a higher affinity than CD28) that are required for activation of both naive T cells and resting clones, whereas PD-1/PD-L1 blockade seems to remove a barrier and enable T cell effector function at the tumor site [116,117]. As the result, ICIs including anti-CTLA-4 and anti-PD-1/PD-L1 Abs were developed to block these inhibitory pathways [118,119,120]. Currently, some of the ICIs including the anti-CTLA-4 agent, ipilimumab, Tremelimumab; anti-PD-1 agents, nivolumab and pembrolizumab; and anti-PDL-1 agent, Atezolizumab, Avelumab, Cemiplimab, Durvalumab, ipilimumab, and atezolizumab [121,122,123] have been approved for the treatment of certain types of cancer [124]. Mechanism action of ICIs are briefly summarized in Figure 5, [124]. Furthermore, in their current review paper, Vaddepally et al. have been briefly reviewed the majority of FDA-approved ICIs per national comprehensive cancer network guidelines [125].

Even though a promising clinical data was obtained using ICIs, still, it shows certain limitations including an occurrence of immune-related adverse events, low response rate, and acquired resistance which is expected to be improved using NPs.

**Figure 6:**
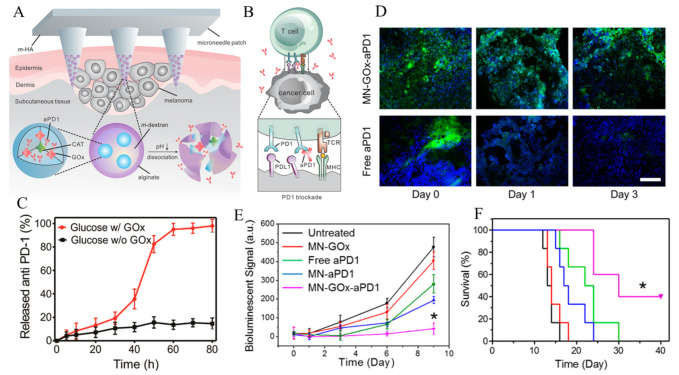
(**A**) Scheme of aPD1 delivery via microneedle (MN) patch, (**B**) Mechanism action of aPD1 to activate T-cell, (**C**) aPD1 release (%) from the MN patches in the presence of 100 mg/dL glucose solution at 37 °C, (**D**) Immunofluorescence staining of tumors treated with MN-GOx-aPD1 or free aPD1 at different time points (green: aPD1, blue: nucleus), (**E**) Bioluminescence signals vs. time after treatment with different groups, and (**F**) % Survival plot of mice after MN patch-assisted delivery of aPD1 therapy. P value: *, *p* < 0.05. Reproduced with permission from [126]. Copyright 2016, American Chemical Society.

Wang et al. designed pH-sensitive microneedle (MN) patch for the sustained delivery of anti-PD1 (aPD1) (Figure 6) [126]. Glucose oxidase was used to generate acidic environments by converting glucose to glucuronic acid, leading to NPs self-dissociation, which in turn facilitates sustained aPD1 releases. The authors found that, at the same dose, pH-sensitive MN patch induces more immune responses compared to non-sensitive MN or free aPD1 using B16F10 mouse melanoma model. Furthermore, the author demonstrated that the aCTLA-4 and aPD1 co-loaded in MN patch shows synergistic effects.

**Figure 7:**
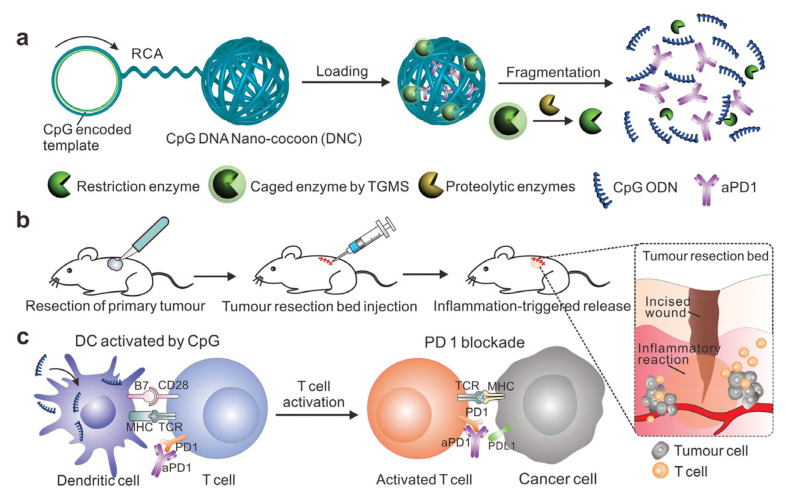
Schematic illustration of (**a**) aPD1 and caged restriction enzyme loaded DNA nanococoon (DNC), (**b**) In vivo tumor immunotherapy after primary tumor resection, local injection, and treatment of DNC-based delivery system and (**c**) Activation of DCs by CpG which in turn activates T cell response with aPD1 for PD 1 blockade. Reproduced with permission from [127]. Copyright 2016, WILEY-VCH Verlag GmbH & Co. KGaA, Weinheim.

Similarly, Wang et al. have been designed inflammation-triggered CpG DNA-based “nano-cocoons” for co-delivery of anti-PD-1 Ab and CpG oligodeoxynucleotides (CpG ODNs) (Figure 7) [127]. The author reported that in comparison to free CpG nucleotides and aPD1, bioresponsive controlled release of CpG and aPD1 showed a considerable immune response and better therapeutic efficacy.

Several researchers reported that patients with advanced cancer poorly respond to PD1/PD-L1 inhibitory therapy due to low TAA expression [128,129]. Epigenetic alteration like DNA hypermethylation, which is commonly seen at TAA promoter regions, plays an essential role in immune evasion of cancer cells during tumorigenesis [130]. Hence, epigenetic modulators, such as hypomethylation agents (HMAs), play a key role in the induction of TAA expression, which in turn increase antitumor immune response [131]. Ruan et al. synthesized a pH and reactive oxygen species (ROS) sensitive bioresponsive gel depot for co-delivery of aPD1 and Zebularine (Zeb), HMA [132]. The author reported that combination therapy enhances the immunogenicity of cancer cells and plays a crucial role in converting immunosuppressive TME.

Preclinical animal studies using cancer nanovaccines, nanoformulated TAA, or tumor-specific neoantigens, revealed promising therapeutic efficacy [133]. However, the clinical use of these nanovaccines has been limited due to immune evasion and suppression in the TME [134,135]. Some literature showed that high expression of immune checkpoints such as PD-L1 is responsible for the occurrence of tumor resistance to vaccine-mediated immune responses. Hence, it possible to overwhelm this limitation by combining with ICIs such as anti-PD-1, anti-PD-L1 or anti-CTLA4 Ab.

**Figure 8:**
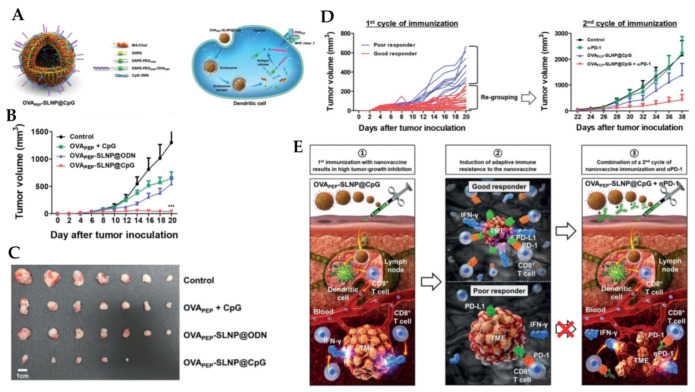
(**A**) Scheme and mechanism action of OVA_PEP_-SLNP@CpG nanovaccine, (**B**) Therapeutic efficacy of OVA_PEP_-SLNP@CpG nanovaccine in an established tumor model, (**C**) representative image of tumors. Scale bar = 1 cm, (**D**) First cycle and second cycle of immunization, (**E**) Overall process of sequential and timely combination strategy between cancer nanovaccine. *p* < 0.001, ***, *p* < 0.05, *, Reproduced with permission from [136]. Copyright 2020, Wiley-VCH Verlag GmbH & Co. KGaA, Weinheim.

Kim et al. developed a small lipid nanoparticle (SLNP)-based nanovaccines embedded with antigen/adjuvant (OVA_PEP_-SLNP@CpG), Figure 8 [136]. Synthesized nanovaccine showed high potent antitumor efficacy in both prophylactic and therapeutic E.G7 tumor models but induced T cell exhaustion by increasing PD-L1 expression, leading to tumor recurrence. However, by using mice that showed a good therapeutic response after the first cycle of immunization with the nanovaccine the author underwent a second cycle together with anti-PD-1 therapy. Their result revealed tumor relapse of suppressed, treatment sequence, and the timing of each modality is crucial in order to enhance antitumor efficacy using combinations of nanovaccines with ICIs.

Similarly, Fontana et al. have designed and assessed biohybrid nanovaccines in combination with anti-CTLA4 antibody [137]. The author observed, an increased activation of APCs and increased priming of CD8^+^ T cells after nanovaccine injection. Most interestingly, treatment efficacy was increased (87.5% of the animals responding, with 2 remissions) in the co-administration (nanovaccine with anti-CTLA4 antibody) compared to the checkpoint inhibitor alone in the B16.OVA model.

#### 2.1.5. Nanoparticles as the Direct Immunomodulators

Immunomodulatory compounds such as cytokines, monoclonal antibodies and adjuvants have been used to reshape the TME and to initiate anti-tumor immunity; although there are certain limitations such as therapeutic efficacy and unwanted side effects during systemic administration, to use in clinics [138].

Immunomodulatory NPs can readily improve the therapeutic effects by enhancing immune stimulation and minimizing off-target side effects. As the result, more research works are undergoing to understand the mechanisms of NPs-Immuno-interactions which is highly important to know the immunomodulating potential of NPs, as the immunostimulating or as immunosuppression [139]. The function of NP in the immunomodulation depends on several factors that are intrinsic to NPs, such as surface chemistry, charge, size, and shape, besides extrinsic factors such as route of administration [140].

Several researchers have widely explored the immunomodulating effects of both polymeric and inorganic NPs [28,141,142]. Different evidence suggests that the immune system cells interact with NPs through Toll-like receptors (TLRs) [143,144]. TLRs are transmembrane proteins, expressed on APCs such as DCs and macrophages, which recognize specific molecular patterns that act as danger signals to the immune system [145]. Depending on the type of receptor and the type of stimuli, TLR engagement plays a great role both in the innate and adaptive immune response by altering several gene expressions.

Inorganic NPs such as Gold nanoparticles (AuNP), Titanium nanoparticles (TiNPs), iron nanoparticles (FeNPs), Zinc nanoparticles (ZnNPs), and silver nanoparticles (AgNPs) are the most stable and promising particles to modulate immune systems [146,147,148].

Vasilichin et al. investigated the influence of metal oxide NPs on innate immunity by testing TLR-4/6 mRNAs in the human monocyte cell line [149]. They found that all studied NPs activated TLR-6 expression, while AlOOH enhanced both TLR-4 and -6 expression.

Moreover, in human peripheral blood mononuclear cells, the administration of AuNPs activates immune-related genes depends on its physicochemical properties [150]. Lee et al. reported that gold nanorods (GNRs) and SiO_2_-coated GNRs has a tendency to penetrate into macrophages to induce the release of inflammatory mediators (calcium (Ca), hydrogen peroxide, nitric oxide (NO), cytokines, prostaglandins, etc.) and the activation of immune response genes [151]. Both GNRs and SiO_2_-coated GNRs have an immunostimulatory property to reinforce immune reactions via calcium—transcription factors pathway.

Fallarini et al. synthesized mono- and disaccharides coated AuNPs with a particle size of ~2 and 5 nm [152]. Their in vitro results showed that synthesized NPs initiate the immune response by activating the macrophages. However, unlike monosaccharide coated AuNPs, disaccharide coated tends to induce T cell proliferation and an increase in IL-2 levels. According to this report, the immunoactivity is strongly dependent on size, 5 nm AuNPs perform far better than 2 nm ones.

Lin et al. also reported that CpG modified AuNP induced macrophage and DC tumor infiltration and suppresses tumor growth compared with free CpG [153]. Similarly, Ahn et al. also reported that AuNP facilitates tumor-associated self-antigen delivery to DC and then activates the cells to facilitate cross-presentation and induce antigen-specific cytotoxic T cell responses [154].

AgNPs also trigger inflammatory reactions cascade involving the activation of macrophages, neutrophils, and helper T cells [155]. Subsequently, AgNPs enhance the expression of numerous types of cytokines [156,157]. Furthermore, different researchers have been investigated the effect of AgNPs as the immunological adjuvant using both in vitro and in vivo studies [158,159].

Xu et al. have investigated an adjuvant effect of AgNPs [160]. The in vivo result showed that serum antigen-specific IgG and IgE levels were increased, showing that AgNPs elicited CD4^+^-mediated immune response. After 48h treatment with AgNPs, both the number of leukocytes and levels of cytokines TNF-α and IFN-γ was increased in abdominal lavage fluid of mice. Furthermore, the expression of the MHC complex class II molecule on the surface of peritoneal macrophages was significantly increased.

In addition, NPs can be designed as artificial APCs (aAPCs), that express surface features, that can activate immune cells or modulate the expression of pro- or anti-inflammatory genes [161,162]. This immunomodulatory behavior of NPs can enhance the therapeutic response of injected NPs by directly generating cytotoxic T cells. For example, Mandal et al. designed biocompatible and less-toxic anti-CD3 antibodies-modified artificial APCs based on poly (isocyano peptide) [163]. They found that synthesized aAPCs induce a more robust T cell response in comparison to free antibodies or PLGA particles. Similarly, Kosmides et al. designed and investigated the synergy between a PLGA-based aAPC and an aPD1 mAb [164]. Their in vitro results revealed that the combination of antigen-specific aAPC and aPD1 mAb induced IFN-γ secretion by CD8^+^ T cells. In addition, in vivo results showed that combination treatment synergistically inhibits tumor growth, while either treatment alone had no effect.

## 3. Clinical Translation of Nano-Immunotherapy

In the last few decades, several researchers have deeply explored a regulatory mechanism of antitumor immunity, particularly the immune checkpoint pathways, which lays a basic foundation for the invention of ICIs, that have revolutionized cancer treatment [165,166]. However, different literature showed that the activity of ICIs as monotherapy is not satisfactory for all cancer patients [167]. To address this clinical challenge, the different researchers tried to combine NPs with immunotherapeutic agents or conventional cancer treatment with ICIs [168,169]. Several kind of the literature showed that, conventional cancer treatments such as chemotherapy, photodynamic therapy, and radiotherapy can initiate the immune system to elicit a specific antitumor immunity, due to its ability to induce immunogenic cell death, in addition, to directly killing cancer cells, which can induce a release of certain damage-associated molecular patterns (DAMPs) that can activate APCs [170]. Activated APCs in turn phagocytose dying tumor cells and present tumor antigens to initiate T cell responses [171]. By taking this into consideration, NPs-based chemotherapeutic agents or photosensitizer delivery can be used to exploit the ICD inducing properties to achieve potent antitumor efficacy in combination with immunotherapeutic agents such as ICIs [172]. Most importantly, NPs based drug delivery can enhance selective target delivery and reduce off-target cytotoxicity of chemotherapeutic or immunotherapeutic agents which in turn extends the therapeutic index, especially for combination therapy.

As briefly discussed above, targeting APCs, cancer cells or TME clearly indicates that NPs significantly improved the therapeutic efficacy of immunotherapeutic agents. Based on the progress made so far, nano-immunotherapy has been achieving remarkable results, some of them were approved by the FDA, and the majority of them are in the preclinical stage, for the treatment of cancer. The first nano-immunotherapy approved for the treatment of advanced triple-negative breast cancer (TNBC) was Atezolizumab (Tecentriq^®^), an ICI against PD-L1, in combination with albumin-bound paclitaxel NP (nab-paclitaxel) [173,174]. The result showed that atezolizumab plus nab-paclitaxel significantly prolonged progression-free survival (PFS) compared to nab-paclitaxel in the intent-to-treat population and the PD-L1 positive subgroup.

Furthermore, Hensify^®^/NBTXR3, 50 nm crystalline hafnium oxide (HfO_2_) NP, received European market approval (CE Mark) in April 2019 for the treatment of locally advanced soft tissue sarcoma in combination with radiation therapy [175]. Hensify^®^ is designed by Nanobiotix to physically destroy tumors and stimulate the immune system locally [176]. Nanobiotix is also running several clinical trials and has received US FDA approval to launch a combination trial with NBTXR3 and PD-1 antibodies to treat lung cancer (NCT03589339).

Similarly, the multicentre, randomized, open-label, phase 3 trial study was conducted as a first-line treatment for metastatic non-squamous non-small-cell lung cancer (IMpower130, NCT02367781) using Atezolizumab in combination with carboplatin plus nab-paclitaxel chemotherapy compared with chemotherapy alone [177]. The result revealed that there were significant improvements in median overall survival (OS), 18.6 months in the atezolizumab plus chemotherapy group, 13.9 months in the chemotherapy group, median PFS 7.0 months in the atezolizumab plus chemotherapy group, and 5.5 months in the chemotherapy group.

Furthermore, there is the first randomized phase 3 JAVELIN Ovarian 200 trial (NCT02580058) study which is designed to demonstrate that Avelumab (human immunoglobulin G1 anti-PD-L1 monoclonal antibody) alone or in combination with Pegylated liposomal doxorubicin (PLD) is superior to PLD alone in prolonging OS in patients with platinum-resistant/platinum refractory ovarian cancer [178]. The results revealed that PLD combined with avelumab slightly improved OS (15.7), PFS (3.7), and objective response rate (ORR) (13.3) compared to either PLD (13.1, 3.5, and 4.2 for OS, PFS, and ORR, respectively) or avelumab (11.8, 1.9, and 3.7 for OS, PFS, and ORR, respectively) alone (Reference: ClinicalTrials.gov; NCT0258005). In addition, RNA formulated NPs alone or in combination with immunotherapeutic agents, such as ICIs, were also explored and the majority of them are under clinical trials as listed in Table 1. Moreover, in his recent review, Yang Shi was briefly reviewed several studies that are FDA approved or under clinical trials using nano-immunotherapy, such as NPs albumin-bound paclitaxel, Pegylated liposomal doxorubicin, mRNA nanovaccines, and WDVAX [179].

In summary, several clinical and preclinical study results demonstrate that NPs are highly important in immunotherapy as the delivery of immunotherapeutic agents or as the direct immunomodulators. However, due to the multifactorial nature of cancer-immune interactions, identifying unique biomarkers are crucial to designing multifunctional NPs (i.e., which have a diagnostic and theranostic application). Hence, in order to design a novel biomarker-guided multifunctional and biocompatible NPs to enhance the efficacy and to promote clinical translation of nano-immunotherapy, a unique biomarker must be identified to distinguish which immune-activating or immunosuppressive cells or pathways are targeted.

## 4. Conclusions

Cancer immunotherapy is emerging as a beneficial tool for cancer treatment by activating the immune system to produce antitumor effects. However, there are some limitations to immunotherapy including poor therapeutic targeting and unwanted side effects. Currently, one of the emerging tracks in this field area is NPs-based immunotherapy which has a considerable potential to solve limitations related to immunotherapy. NPs plays a great role in cancer immunotherapy as the carrier of immunotherapeutic agents and as the direct immunomodulator. NPs based delivery of immunotherapeutic agents offers a great opportunity to minimize unwanted cytotoxicity through controlled release, dose-sparing, or enhanced tumor targeting capabilities. Hence, in the near future, as our knowledge enhanced to understand the detailed molecular mechanism of NPs-immune interaction, NP-based therapies will revolutionize and place NP-based immunotherapy at the forefront of immune-modulating therapeutics.

## Figures and Tables

**Table 1:** FDA approved nano-Immunotherapy and studies under clinical trials to treat cancer [180,181,182].

Compound Name	Formulation Description	Mechanism of Action	Clinical Trials	Approved by the FDA	Ref
RNA-LPX (Lipoplex^®^)	RNA-lipoplexes	DC maturation, T cell response	Phase I (2016)		[82]
MRX34	miRNA-34a-loaded liposome	Downregulation of immune evasion tumor genes	Phase I (2016)		[183]
mRNA-4157	mRNA-4157 encapsulated in Lipids	induce neoantigen specific T cells and associated anti-tumor responses.	Phase I (2019)		[184]
Ferumoxytol (Ferahem^®^)	Iron oxide nanoparticles (IONP)	M2 Macrophage polarization to M1-like		Yes, for anemia and kidney diseases	[185]
PTX-LDE	Paclitaxel-loaded lipid core NPs	DC maturation	Phase II (2017)		[186,187]
Anti-EGFR-IL-dox	Doxorubicin-loaded anti-EGFR immunoliposomes	Block EGFR-mediated growth signaling and induce immunogenic cell death	Phase II (2016)		NCT02833766
JVRS-100	Cationic liposome incorporating plasmid DNA complex	Immune system stimulation	Phase I (2016)		NCT00860522
NBTXR3	Hafnium oxide nanoparticles in combination with anti-PD1	Enhance tumor cell death via electron production, induce immunogenic cell death leading to activation of the immune system	Phase I (2019)		[188], NCT03589339
